# Simultaneous Quantification of Ellagitannins and Related Polyphenols in *Geranium thunbergii* Using Quantitative NMR

**DOI:** 10.3390/molecules23061346

**Published:** 2018-06-04

**Authors:** Februadi Bastian, Yurie Ito, Erika Ogahara, Natsuki Ganeko, Tsutomu Hatano, Hideyuki Ito

**Affiliations:** 1 Faculty of Health and Welfare Science, Okayama Prefectural University, 111 Kuboki, Soja, Okayama 719-1197, Japan; februadi@unhas.ac.id (F.B.); yurieng8@icloud.com (Y.I.); ganeko@fhw.oka-pu.ac.jp (N.G.); 2 Division of Pharmaceutical Sciences, Okayama University Graduate School of Medicine, Dentistry, Pharmaceutical Sciences, 1-1-1 Tsushima-naka, Kita-ku, Okayama 700-8530, Japan; fraaaan.1441@gmail.com (E.O.); hatano-t@cc.okayama-u.ac.jp (T.H.)

**Keywords:** ^1^H-NMR, quantitative NMR, ellagitannin, *Geranium thunbergii*, geraniin

## Abstract

Compared to commonly employed liquid chromatography-based methods, quantitative nuclear magnetic resonance (qNMR) is a recently developed method for accurate quantification of natural compounds in extracts. The simultaneous quantification of ellagitannins and the related polyphenols of *Geranium thunbergii* were studied using qNMR after a short-term and long-term decoction. The qNMR fingerprint for quantifying ellagitannin was presented in this work. Geraniin was observed in the short-term decoction as a major component while corilagin was the major component of the long-term decoction. An aqueous acetone extract of *G. thunbergii* after long-term decoction was extracted with diethyl ether, ethyl acetate, and *n*-butanol. Corilagin was found as a major constituent in the ethyl acetate and *n*-butanol extracts. Furthermore, the contents of these polyphenols in *G. thunbergii* from six locations in Japan and three locations in China were quantified. The contents of geraniin and corilagin in *G. thunbergii* from Japan were higher than those from China. Our finding raised the possibility that qNMR can be effectively employed as a simple, accurate, and efficient method for quantification of ellagitannins in medicinal plants.

## 1. Introduction

Nuclear magnetic resonance (NMR) has become the leading analytical tool for chemical structure elucidation in numerous fields of industrial and academic research. One advantage for using NMR is that the technique is non-destructive, enabling compound detection from both pure and impure samples without compromising the initial sample [1,2,3]. NMR has the potential to simultaneously provide both qualitative and quantitative information.

Since 1963, NMR spectroscopy has been described as a quantitative measurement [4]. Quantitative nuclear magnetic resonance (qNMR) is founded on the principle that signal intensities of a given NMR spectrum are directly proportional to the molar amount of that nucleus in the sample, so that qNMR can be a simple and absolute quantification method able to determine the purities of compounds or the absolute content of a compound in the natural source with unit traceability. The past decade has seen an increasing amount of literature on the usefulness of qNMR. The survey by Pauli and co-workers showed that quantification using NMR on pharmaceutical, chemical, and food fields has significantly increased in recent years [5,6]. The usefulness of qNMR is further accelerated by additional advantages, such as the lack of calibration curve requirements, the non-destructive character of the NMR technique, for the lack of special sample preparation requirements, relatively short measurement times, and the possibility of simultaneously quantifying multiple compounds in crude extracts [5,6,7,8,9,10].

The accuracy of a qNMR measurement is one of the key reasons for why this method was employed. Previous studies have established no significant difference between the accuracy of assays conducted using qNMR and other methods, such as HPLC. Huo et al. measured the content of avermectin using HPLC and qNMR. They reported no significant difference between the assay results of these two methods [10]. Similarly, Napolitano et al. plotted the concentration of catechin obtained by HPLC-MS/MS against values obtained using qNMR. Both methods yielded similarly good linear regression correlations of R^2^ > 0.999. No significant difference between HPLC-MS/MS and qNMR methods was found [3]. For these reasons, combined with numerous additional advantages, qNMR might be a powerful tool for the quantification of natural products.

Ellagitannins belong to the class of hydrolysable tannins. They have shown potential health benefits, such as the prevention of advanced glycation end products formation [11], anti-inflammatory effects [12,13], anti-diabetic effects [14], anti-fungal effects [15], and antioxidant effects [16,17]. It is now well established from a variety of studies that over 500 ellagitannins have been discovered [18,19]. *Geranium thunbergii,* a member of the *Geranium* genus, is well known to contain large amounts of ellagitannins. The dried weight of *G. thunbergii* leaves can contain up to 10% of ellagitannins, including geraniin and corilagin. Geraniin contains the typical acyl groups found in ellagitannins, such as galloyl, hexahydroxydiphenolyl (HHDP), and dehydrohexahydroxydiphenolyl (DHHDP) units [20]. Therefore, we selected a suitable candidate for the present study.

Geraniin is the major ellagitannin in *G. thunbergii*, which has long been used as a remedy for intestinal disorders in Japan [20,21]. Geraniin has also been reported to have various biological activities [22]. *G. Thunbergii* is traditionally known in Japan as *gennoshoko* [20]. There are two ways to use *G. thunbergii* medicinally. The dried aerial parts can be brewed with hot water (making a tea) or the dried plant can be boiled in water for one hour. The resulting concoctions are used for the treatment of constipation and diarrhea, respectively.

**Figure 1:**
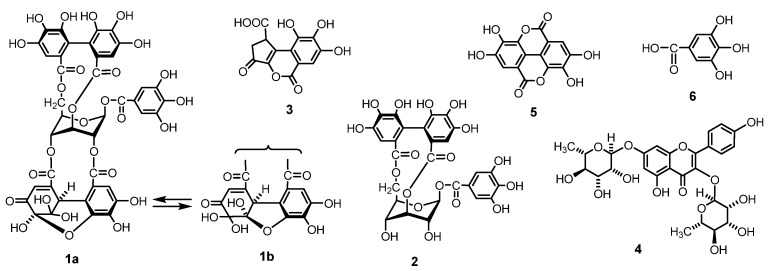
Structures of the main ellagitannins and related polyphenols in *G. thunbergii*.

To the best of our knowledge, no study has yet investigate the quantity of ellagitannin in *G. thunbergii* using qNMR. Most researchers quantifying ellagitannin have utilized chromatographic methods, such as HPLC, UHPLC, and LC-MS/MS [23,24,25]. Analytical methods using HPLC require pure standards for drawing the calibration curve. It is well known that obtaining commercially pure native standards of specific ellagitannins is difficult. For HPLC and other chromatographic studies, an initial purification of the specific ellagitannin is generally required for use in further quantification processes. In addition, large ellagitannin oligomers are more difficult to purify than monomers [26]. In this study, we investigated the application of qNMR to assay the main ellagitannin and the related polyphenol contents (Figure 1) in the short-term and long-term extracts of *G. thunbergii*. The present study also explored the main polyphenol contents in *G. thunbergii* plants cultivated in Japan and China. The contents of the targeted compounds were calculated from the integral of the characteristic anomeric or aromatic proton signals against that of an internal standard.

## 2. Results

### 2.1. HPLC Analysis of Geraniin and the Related Polyphenols in G. thunbergii

Quantification of geraniin using HPLC has several weaknesses. Geraniin, belonging to the dehydroellagitannin family, usually forms an equilibrium mixture of six- and five-membered hemiacetal forms (**1a** and **1b,** respectively) in aqueous solutions. Thus, methanol adducts with methoxyl groups at hemiacetal in geraniin were produced in methanol solution subjected to HPLC resulted in showing multiple or broad peaks in HPLC profile. [27,28].

**Figure 2:**
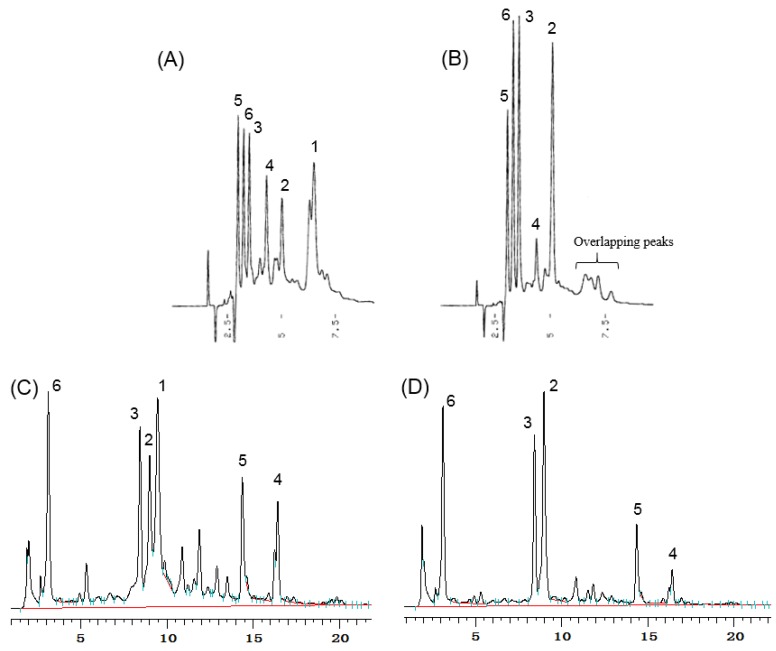
HPLC profiles for the extracts of *G. thunbergii*: (**A**) short-term decoction on normal phase HPLC; (**B**) long-term decoction on normal phase HPLC; (**C**) short-term decoction on reversed phase HPLC; and (**D**) long-term decoction on reversed phase HPLC.

In this study, problems of coelution on HPLC during the analysis of geraniin were found. The HPLC profiles of *G. thunbergii* extracts after brewing in hot water showed the geraniin (**1**) peak. After boiling for 1 h, geraniin was completely hydrolyzed to corilagin (**2**), brevifolincarboxylic acid (**3**), ellagic acid (**5**), and gallic acid (**6**). However, after boiling for 1 h, selected signals at the same retention time to geraniin remained on the chromatograph in normal phase HPLC, implying that other compounds overlapped with the retention time signal corresponding to geraniin (Figure 2). Furthermore, ellagic acid, gallic acid, and brevifolincarboxylic acid elute closely on normal phase HPLC. The reversed phase HPLC profile also revealed that brevifolincarboxylic acid, geraniin, and corilagin all eluted close together. This HPLC coelution phenomenon is one of the reasons why we began to explore other methods to quantify ellagitannins and the related polyphenols.

### 2.2. ^1^H-NMR Fingerprint

There are two critical issues for determining the type of solvent to employ: the ability to dissolve the compounds of interest and signal separation. Dimethyl sulfoxide (DMSO)-*d*_6_ was a very good choice for solvating ellagitannins, but it did not provide sufficient ^1^H-NMR signal separation.

Despite the addition of CF_3_COOD-D_2_O and employing variable temperature techniques, sufficient peak separation could not be obtained. Alternatively, acetone-*d*_6_ could provide adequate ^1^H-NMR signal separation, but it was inadequate at dissolving some of the target compounds. We could not obtain valuable data in the solvent with good solubility such as methanol-*d*_4_, since necessary signals corresponding to some of candidate compounds were overlapped with a signal of HDO in the solvent. On the other hand, acetone-*d*_6_ has less area overlapping with HDO. Furthermore, a small amount of D_2_O was added to raise the solubility and CF_3_COOD was added to provide sharp signals due to polyphenols. With peak separation being the most important factor in qNMR analysis, acetone-*d*_6_ was selected and D_2_O and CF_3_COOD were added to obtain a final ratio of 70:25:5, respectively. This solvent ratio resulted in good ^1^H-NMR peak separation.

**Figure 3:**
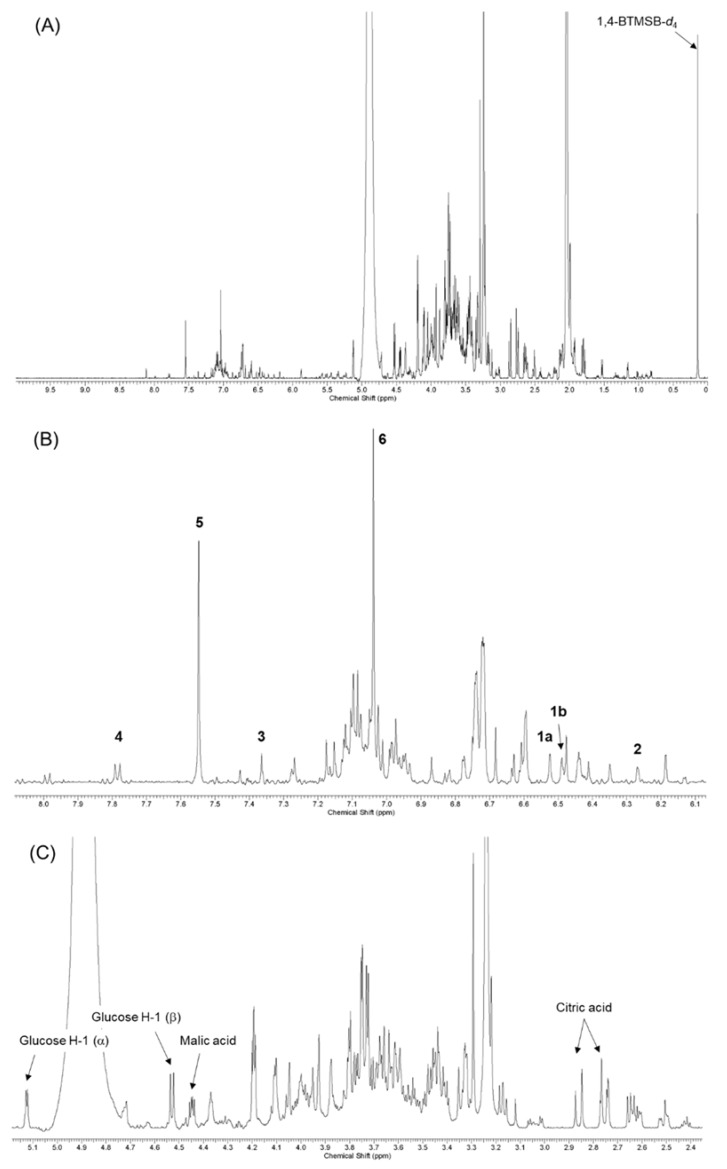
^1^H-NMR spectra {600 MHz, acetone-*d*_6_-CF_3_COOD-D_2_O (70:25:5)} of the short-term decoction of *G. thunbergii*: (**A**) entire spectrum; (**B**) expansion of the low-field region; and (**C**) expansion of the high-field region.

To develop the ^1^H-NMR fingerprints indicative of geraniin and its metabolites, sugar, and organic acids, ^1^H-NMR spectrum analyses of the individual compounds were carried out. The indicative signals of the candidate compounds for qNMR are presented in Table 1 and Figure 3. Based on the integrations of these signals, the contents of ellagitannin, and the related compounds, in various extracts of *G. thunbergii* were quantified by the method described in Section 4.4.

### 2.3. Short-Term and Long-Term Decoctions

Based on the selected peaks for qNMR in Table 1, we evaluated the composition differences of the target polyphenols in *G. thunbergii* obtained from extraction in both short-term and long-term decoctions. Extraction by short-term decoction showed geraniin as the predominant ellagitannin and the related polyphenols (62.0% of the total polyphenols). In contrast, corilagin (corresponding to the hydrolysate of geraniin) was a major extraction compound from long-term decoction. During short-term decoction, the amount of corilagin was only 13.2%. After long-term decoction, the amount of corilagin increased to 49.0% (Table 2). Additionally, the percentages of gallic acid, ellagic acid, and brevifolincarboxylic acid also increased. However, geraniin was not detected and no significant difference was found between the amount of kaempferitrin before and after long-term decoction. These results indicate that geraniin was hydrolyzed to corilagin, gallic acid, ellagic acid, and brevifolincarboxylic acid after 1 h of decoction.

**Figure 4:**
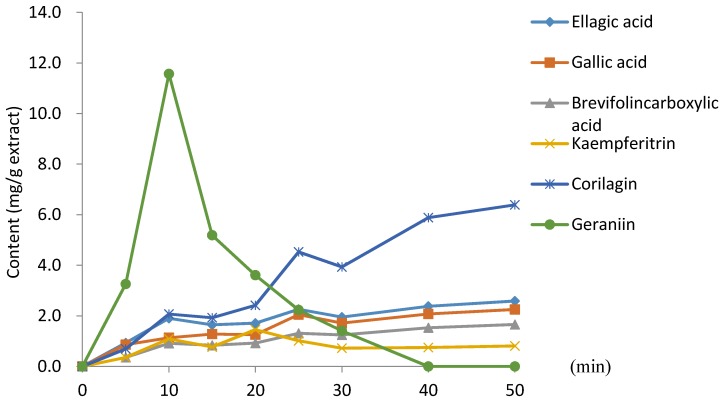
Change in main polyphenol content during *G. thunbergii* decoction.

Figure 4 presents a graph of the change in main polyphenol content during 50 min of decoction. Geraniin was found to increase until 10 min into decoction; thereafter, it decreased sharply. In contrast, the amount of corilagin increased steadily over the entire 50 min decoction [29]. The amounts of brevifolincarboxylic acid, gallic acid, and ellagic acid also increased steadily.

### 2.4. The Main Polyphenol Contents in each Extract of G. thunbergii

**Figure 5:**
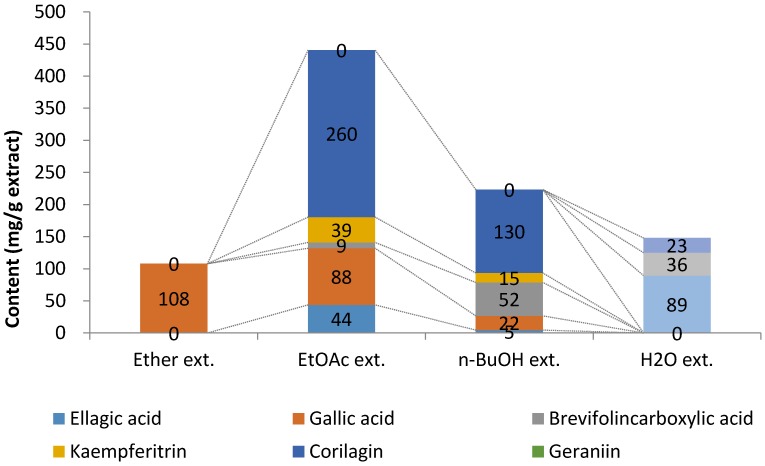
The main polyphenol and organic acid contents in each extract from the long-term decoction of *G. thunbergii.*

Next, the extract obtained from long-term decoction of *G. thunbergii* was successively extracted with diethyl ether, ethyl acetate, and water-saturated *n*-butanol. The ether, ethyl acetate, *n*-butanol, and water-soluble extracts were measured for main polyphenols and organic acid content using the qNMR method. The ether extract contained gallic acid exclusively. Gallic acid was also found in the ethyl acetate extract and *n*-butanol extract. Corilagin accounted for half of the extracts in ethyl acetate and *n*-butanol. (Figure 5). These data indicate that ellagitannins, such as corilagin, could be effectively extracted by ethyl acetate and *n*-butanol.

### 2.5. Amount of Main Polyphenols in G. thunbergii Cultivated in Japan and China

We estimated the amount of main polyphenols in *G. thunbergii* from nine cultivates in Japan and China using our qNMR method. All samples were extracted with 70% aqueous acetone. As shown in Table 3, *G. thunbergii* in Japan contained geraniin in the range of 3.49 to 7.41 mg/g (average: 5.21 mg/g) whereas geraniin content in *G. thunbergii* from Zhejiang province in China was in the range of 0.44 to 2.82 mg/g (average: 1.61 mg/g). The amounts of corilagin, gallic acid, ellagic acid, and brevifolincarboxylic acid were very similar between the Japanese and Zhejiang cultivates. However, kaempferitrin was found in the Japan cultivates in the range of 0.13 to 0.56 mg/g (average: 0.2 mg/g) while the three cultivates from Zhejiang did not contain kaempferitrin.

## 3. Discussion

We developed ^1^H-NMR fingerprints for quantifying ellagitannins, especially in *G. thunbergii* extract. Several specific proton signals from these ellagitannins were identified for geraniin, corilagin, corilagin, ellagic acid, brevifolincarboxylic acid, and organic acids, such as malic acid and citric acid. Table 1 and Figure 3 show the chemical shift used for further quantifying the *G. thunbergii* extracts in this study.

Furthermore, we evaluated the quantification of polyphenols using our qNMR assay in short-term and long-term decoctions of *G. thunbergii*. Geraniin was the major compound (62.0%) in the short-term decoction extract and corilagin was the major compound (49.0%) from the long-term decoction extract (Table 2). The amount of geraniin reached a maximum after 10 min of extraction. Geraniin was not observed by 40 min thereafter due to hydrolysis. Instead, corilagin became the dominant compound.

In Japan, there are two methods of using *G. thunbergii* as a folk medicine. The short and long decoctions are used for treating constipation and diarrhea, respectively. Our findings suggested that both geraniin and corilagin possess the ability to treat gastrointestinal diseases, but that they may show distinct functions. It might be speculated that geraniin has an ability to treat constipation while corilagin plays a key role in diarrhea treatment.

Fractionation of the long-term decoction showed that corilagin was obtained in the ethyl acetate and *n*-butanol fractions. Based on our qNMR method, corilagin was the major compound in both fractions, obtained at 59% and 58% in ethyl acetate and *n*-butanol, respectively. Among the solvents tested (diethyl ether, ethyl acetate, *n*-butanol, and water), ethyl acetate and *n*-butanol were found suitable for extraction of the main ellagitannin. In addition, our data indicated that only gallic acid could be extracted by diethyl ether. Malic acid, citric acid, and glucose were all obtained in the water-soluble portion (Figure 5).

The main ellagitannin contents of *G. thunbergii* extracts from plants cultivated in Japan and Zhejiang province in China are presented in Table 3. We found that the *G. thunbergii* cultivates from Japan contained higher levels of geraniin and corilagin than those from Zhejiang. Overall, the polyphenol content of cultivates from Japan were higher than those from Zhejiang. Furthermore, the six cultivates of *G. thunbergii* from Japan contained kaempferitrin, whereas the three cultivates from Zhejiang did not contain kaempferitrin. Differences in the content of these polyphenols in *G. thunbergii* may be affected by soil and weather conditions.

All samples were directly quantified using qNMR after the extracting solvents were removed. The advantages of qNMR include: accuracy, simplicity, and speediness. This is because the initial separation of target analytes into pure compounds is unnecessary. Therefore, further research should be undertaken to investigate other specific chemical shifts from other ellagitannins to enrich the number of unique qNMR ellagitannin identifier peaks.

## 4. Materials and Methods

### 4.1. Plant Material

Japanese pharmacopoeia standard products *G. thunbergii* (*gennoshoko*) cultivated in Japan and China were purchased from Japanese companies, Kokumin, Konishi, Kojuma, Daiko, Eidai, Matsuura, Japan Health, and Uchida.

### 4.2. HPLC Analysis

The normal phase HPLC system consisted of a pump (Jasco PU-980), UV-Vis detector (Shimadzu SPD-6A, Kyoto, Japan), and a column (YMC-Pack SIL A-003, 4.6 mm I.D × 250 mm). The mobile phase consisted of oxalic acid (450 mg/L) in *n*-hexane:methanol:tetrahydrofuran:formic acid (55:33:11:1, *v*/*v*/*v*/*v*, respectively). The extract (5 μL) was injected into the HPLC and delivered at a flow rate of 1.5 mL/min. The detection wavelength was set to 280 nm. The reversed phase HPLC system consisted of pump (Hitachi L-2130, Tokyo, Japan) equipped with a diode array detector (Hitachi L-7455). The separation employed an Intersustain C18 column (GL Science, 5μm, 4.6 mm I.D. × 150 mm) at 40 °C. The mobile phase consisted of eluent A (H_2_O:acetonitrile:formic acid, 90:5:5 *v*/*v*/*v*) and eluent B (H_2_O:acetonitrile:formic acid, 50:45:5, *v*/*v*/*v*). The eluent was programmed as follows: 0 min 0% B; 30 min. 100% B; 30.1–45 min 0% B. The flow rate of the mobile phase was set at 1.0 mL/min.

### 4.3. NMR Spectroscopy

All NMR analyses were carried out on a Varian NMR System 600PS (Palo Alto, CA, USA) operating at 600 MHz for ^1^H-NMR. The measurement parameters were as follows: pulse flip angle, 90°; number of acquisitions, 8; acquisition time, 4 s; number of points, 48,077; delay time, 60 s; spin, off; temperature, room temperature.

### 4.4. qNMR Analysis

70% aqueous acetone extracts of the short-term and long-term decoction (20 mg) of *G. thunbergii* were dissolved in acetone-*d*_6_:D_2_O:CF_3_COOD (70:25:5, *v*/*v*/*v*) (1 mL) + 1,4-BTMSB-*d*_4_ (0.1 mg) (Fujifilm, Wako Pure Chemical Corporation, Tokyo, Japan). 1,4-BTMSB-*d*_4_ (99.9% purity) was used as an internal standard. Sample quantification was performed according to Equation (1):

(1) $$P_{sample}=\;\frac{I_{sample}/H_{sample}}{I_{1,4-BTMSB-d4}/H_{1,4-BTMSB-d4}}\times \frac{M_{sample}/W_{sample}}{M_{{}_{1,4-BTMSB-d4}}/W_{{}_{1,4-BTMSB-d4}}}\times P_{{}_{{}_{1,4-BTMSB-d4}}}$$

where: *P_sample_* = purity of desired compound; *P*_1,4-*BTMSB-d*4_ = 99.9%; *I_sample_* = area measured by integration of the desired compound on the ^1^H-NMR spectrum; *I*_1,4-*BTMSB-d*4_ = 100; *H_sample_* = number of hydrogen(s) of the desired signal of compound; *H*_1,4-*BTMSB-d*4_ = 18; *M_sample_* = molar mass of the desired compound; *M*_1,4-*BTMSB-d*4_ = 226.5; *W_sample_* = weight of sample (20 mg); *W*_1,4-*BTMSB-d*4_ = 0.1 mg. To determine the weight of the compound (*m_compound_*) in the sample, an absolute equation was used (Equation (2)):

(2) $$m_{compound}=\;\frac{P_{sample}}{P_{1,4-BTMSB-d4}}\times W_{sample}$$

### 4.5. Short-Term and Long-Term Decoction

The short-term decoction was performed by brewing 10 g of dried *G. thunbergii* in 600 mL of boiling water for 1 min. The mixture was then filtered and the obtained liquid was concentrated to dryness on a rotary evaporator to obtain 2.2 g of dried filtrate. The long-term decoction was performed by boiling 10 g of dried *G. thunbergii* in 600 mL water until the water was reduced to 300 mL. After filtration, the filtrate was dried on a rotary evaporator to obtain 0.34 g of extract. These methods imitated the traditional Japanese methods used to treat gastrointestinal diseases with *G. thunbergii*.

The long-term decoction fractionation was carried out by extracting 2.2 g of the dried extract sequentially using diethyl ether, ethyl acetate, *n*-butanol, and water (250 mL for each solvent). The extracts obtained from each solvent were: diethyl ether, 44.6 mg; ethyl acetate, 173.1 mg; *n*-butanol, 269.1 mg; and water 1.72 g.

## 5. Conclusions

Our data indicated that the qNMR method can be used to perform accurate, simple, and rapid analysis of target analyte content without the need for intricate separation steps or authentic materials for calibration. qNMR is a powerful tool for quantification of ellagitannins that have an anomeric center or can equilibrate in solution. Therefore, extensive research on various ellagitannins in medicinal plants and food sources is necessary to enrich the qNMR database of unique marker signals for ellagitannin quantification.

## Figures and Tables

**Table 1:** Specific ^1^H-NMR data of main ellagitannins and the other compound of *G. thunbergii* for quantitative nuclear magnetic resonance (qNMR) analysis {600 MHz, acetone-*d*_6_-CF_3_COOD-D_2_O (70:25:5)}*.*

Compounds	MW	^1^H-NMR Data
Geraniin (**1**)	952	δ 6.54 (**1a** form glucose H-1; 1H, br s) δ 6.51 (**1b** form glucose H-1; 1H, br s)
Corilagin (**2**)	634	δ 6.30 (glucose H-1; 1H, br s)
Brevifolincarboxylic acid (**3**)	292	δ 7.36 (aromatic proton; 1H, s)
Kaempferitrin (**4**)	578	δ 7.78 (B-ring H-2′,6′; 2H, d, *J* = 9.0 Hz)
Ellagic acid (**5**)	302	δ 7.55 (2H, s)
Gallic acid (**6**)	170	δ 7.04 (2H, s)
Glucose	180	δ 4.52 (β-form H-1; 1H, d, *J* = 8.4 Hz) δ 5.11 (α-form H-1; 1H, d, *J* = 3.6 Hz)
Malic acid	134	δ 4.44 (1H, dd, *J* = 4.2, 7.2 Hz)
Citric acid	210	δ 2.85 (1H, d, *J* = 15.6 Hz)

**Table 2:** % composition relative to the amount of total polyphenols in the short-term and long-term decoction extracts of *G. thunbergii*.

Main Polyphenols	Contents (%)	
	Short-Term Decoction	Long-Term Decoction
Geraniin (**1**)	62.0	0.0
Corilagin (**2**)	13.2	49.0
Brevifolincarboxylic acid (**3**)	5.1	13.1
Kaempferitrin (**4**)	6.0	5.4
Ellagic acid (**5**)	6.5	16.1
Gallic acid (**6**)	7.2	16.4

**Table 3:** The amounts of main polyphenols in *G. thunbergii* cultivated in Japan and China (mg/g dried weight).

Polyphenols	Japan						China		
	Fukushima	Nagano 1	Nagano 2	Nagano 3	Hyogo	Miyazaki	Zhejiang 1	Zhejiang 2	Zhejiang 3
Geraniin (**1**)	3.49	4.15	4.23	3.57	8.65	7.14	2.82	1.57	0.44
Corilagin (**2**)	0.65	0.43	0.61	0.23	0.53	0.43	0.49	0.23	0.17
Brevifolincarboxylic acid (**3**)	0.17	0.14	0.25	0.07	0.22	0.09	0.12	0.06	0.05
Kaempferitrin (**4**)	0.13	0.25	0.56	0.12	0.36	0.35	0.00	0.00	0.00
Ellagic acid (**5**)	0.33	0.37	0.41	0.23	0.47	0.30	0.28	0.13	0.12
Gallic acid (**6**)	0.21	0.23	0.31	0.21	0.23	0.03	0.14	0.08	0.09

## References

[1] Weber M., Hellriegel C., Rueck A., Wuethrich J., Jenks P. (2014). Using high-performance ^1^H-NMR (HP-qNMR^®^) for the certification of organic reference materials under accreditation guidelines—Describing the overall process with focus on homogeneity and stability assessment. J. Pharm. Biomed. Anal..

[2] Rituerto E.L., Cabredo S., Lopez M., Avenoza A., Busto J.H., Peregrina J.M. (2009). A thorough study on the use of quantitative ^1^H-NMR in rioja red wine fermentation processes. J. Agric. Food Chem..

[3] Napolitano J.G., Godecke T., Lankin D.C., Jaki B.U., McAlpine J.B., Chen S.N., Pauli G.F. (2014). Orthogonal analytical methods for bionical standardization: Determination of green tea catechins by qNMR and LC-MS/MS. J. Pharm. Biomed. Anal..

[4] Jungnickel L., Forbes J.W. (1963). Quantitative measurement of hydrogen types by integrated nuclear magnetic resonance intensities. Anal. Chem..

[5] Pauli G.F., Jaki B.U., Lankin D.C. (2005). Quantitative ^1^H-NMR: Development and potential of a method for natural product analysis. J. Nat. Prod..

[6] Pauli G.F., Godecke T., Jaki B.U., Lankin D.C. (2012). Quantitative ^1^H-NMR. Development and potential of an analytical method: An update. J. Nat. Prod..

[7] Malz F., Jancke H. (2005). Validation of quantitative NMR. J. Pharm. Biomed. Anal..

[8] Godecke T., Yao P., Napolitano J.G., Nicolic D., Dietz B.M., Bolton J.L., Breemen R.B., Farnsworth N.R., Chen S.N., Lankin D.C. (2012). Integrated standardization concept for Angelica botanicals using quantitative NMR. Fitoterapia.

[9] Tanaka R., Shibata H., Sugimoto N., Akiyama H., Nagatsu A. (2016). Appication of a quantitative ^1^H-NMR method for determination of paenol in moutan cortex, Hachimijiogan and Keishibukuryogan. Nat. Med..

[10] Hou Z., Liang X., Du L., Su F., Su W. (2014). Quantitative determination and validation of avermectin B1a in commercial products using quantitative nuclear magnetic resonance spectroscopy. Magn. Reson. Chem..

[11] Ito H., Li P., Koreishi M., Nagatomo A., Nishida N., Yoshida T. (2014). Ellagitannin oligomers and a neolignan from pomegranate arils and their inhibitory effects on the formation of advanced glycation end products. Food Chem..

[12] Ismail T., Sestili P., Akhtar S. (2012). Pomegranate peel and fruit extracts: A review of potential anti-inflammatory and anti-infective effects. J. Ethnopharm..

[13] Piwowarski J.P., Granica S., Zwierzynska M., Stefanska J., Schopohl P., Melzig M.F., Kiss A.K. (2014). Role of human gut microbiota metabolism in the anti-inflammatory effect of traditionally used ellagitannin-rich plant materials. J. Ethnopharm..

[14] Kashchenko N.I., Chirikova N.K., Olennikov D.N. (2017). Agrimoniin, an active ellagitannin from *Comarum palustre* Herb with anti-α-glucosidase and antidiabetic potential in streptozotocin-induced diabetic rats. Molecules.

[15] Valdes J.A., Burboa E., Carbo A.F.A., Aparicio M., Schmidt R.P., Rodriguez R., Aguilar C.V. (2013). Antifungal ellagitannin isolated from *Euphorbia antisyphilitica* Zucc. Asian Pac. J. Trop. Biomed..

[16] Kaneshima T., Myoda T., Nakata M., Fujimori T., Toeda K., Nishizawa M. (2016). Antioxidant activity of *C*-Glycosidic ellagitannins from the seeds and pell of camu-camu (*Myrciaria dubia*). LWT-Food Sci. Technol..

[17] Kahkonen M., Kylli P., Ollilainen V., Salminen J.P., Heinonen M. (2012). Antioxidant activity of isolated ellagitannin from red raspberries and cloudberries. J. Agric. Food Chem..

[18] Okuda T., Yoshida T., Hatano T., Ito H., Quideau S. (2009). Ellagitannins renewed the concept of tannin. Chemistry and Biology of Ellagitannin: An Underestimated Class of Bioactive Plant Polyphenols.

[19] Okuda T., Ito H. (2011). Tannin of constant structure in medical and food plants-hydrolyzable tannin and polyphenols related to tannins. Molecules.

[20] Okuda T., Yoshida T., Hatano T. (1982). Constituents of *Geranium thunbergii* Sieb. *et* Zucc. Part 12. Hydrated stereostructure and equilibration of geraniin. J. Chem. Soc. Perkin Trans..

[21] Ito H., Hatano T., Namba O., Shirono T., Okuda T., Yoshida T. (1999). Constituents of *Geranium thunbergii* SIEB. et ZUCC. XVI) Modified dehydroellagitannins, geraniinic Acids B and C, and phyllanthusiin F. Chem. Pharm. Bull..

[22] Graca V.C., Ferreira I.C.F.R., Santos P.F. (2016). Phytochemical composition and biological activities of *Geranium robertianum* L.: A review. Ind. Crops Prod..

[23] Fischer U.A., Carle R., Kammerer D.R. (2011). Identification and quantification of phenolic compounds from pomegranate (*Punica granatum* L.). Food Chem..

[24] Pinto M.S., Lajolo F.M., Genovese M.I. (2008). Bioactive compounds and quantification of total ellagic acid in strawberries (*Fragaria x ananasa* Duch.). Food Chem..

[25] Moilanen J., Koskinen P., Salminen J.P. (2015). Distribution and content of ellagitannin in Finnish plant species. Phytochemistry.

[26] Baert N., Karonen M., Salminen J.P. (2015). Isolation, characterization and quantification of the main oligomeric macrocyclic ellagitannin in *Epilobium angustifolium* by ultra-high performance chromatography with diode array detection and electrospray tandem mass spectrometry. J. Chromatogr. A.

[27] Hatano T., Yoshida T., Okuda T. (1988). Chromatography of tannins, III. Multiple peaks in high-performance liquid chromatography of some hydrolyzable tannins. J. Chromatogr..

[28] Okuda T., Yoshida T., Hatano T. (1989). New methods of analyzing tannins. J. Nat. Prod..

[29] Okuda T., Mori K., Ishino M. (1979). Constituents of *Geranium thunbergii* SIEB. et ZACC. VIII. Transformation of geraniin upon decoction. Yakugaku Zasshi.

